# A three dimensional anatomical view of oscillatory resting-state activity and functional connectivity in Parkinson's disease related dementia: An MEG study using atlas-based beamforming^☆^

**DOI:** 10.1016/j.nicl.2012.11.007

**Published:** 2012-11-17

**Authors:** M.M. Ponsen, C.J. Stam, J.L.W. Bosboom, H.W. Berendse, A. Hillebrand

**Affiliations:** a Department of Neurology, VU University Medical Center, de Boelelaan 1117, 1081 HV, Amsterdam, The Netherlands; b Department of Clinical Neurophysiology, VU University Medical Center, de Boelelaan 1117, 1081 HV, Amsterdam, The Netherlands; c Department of Neurology, Onze Lieve Vrouwe Gasthuis, Oosterpark 9, 1091 AC, Amsterdam, The Netherlands; d Magnetoencephalography Center, VU University Medical Center, de Boelelaan 1117, 1081 HV, Amsterdam, The Netherlands

## Abstract

Parkinson's disease (PD) related dementia (PDD) develops in up to 80% of PD patients. The present study was performed to further unravel the underlying pathophysiological mechanisms by applying a new analysis approach that uses an atlas-based MEG beamformer to provide a detailed anatomical mapping of cortical rhythms and functional interactions. Importantly, we used the phase lag index (PLI) as a measure of functional connectivity to avoid any biases due to effects of volume conduction.

MEG recordings were obtained in 13 PDD and 13 non-demented PD patients. Beamforming was used to estimate spectral power and PLI in delta, theta, alpha, beta and gamma frequency bands. Compared to PD patients, PDD patients had more delta and theta power in parieto-occipital and fronto-parietal areas, respectively. The PDD patients had less alpha and beta power in parieto-temporo-occipital and frontal areas, respectively. Compared to PD patients, PDD patients had lower mean PLI values in the delta and alpha bands in fronto-temporal and parieto-temporo-occipital areas, respectively. In addition, in PDD patients connectivity between pairs of regions of interest (Brodmann areas) was stronger in the theta band and weaker in the delta, alpha and beta bands.

The added value of the present results over previous studies analysing frequency-specific changes in neuronal activity in PD patients, is the anatomical framework in which we demonstrated a slowing in neuronal activity and a reduction in functional connectivity in PD related dementia. Moreover, this study shows a widespread reduction in functional connectivity between different regions in PDD.

## Highlights

► MEG was recorded in demented (PDD) and non-demented (PD) Parkinson patients. ► Atlas-based MEG beamformer was used for anatomical mapping of neuronal activity. ► Slowing of neuronal activity (power) in PDD compared to PD. ► Reduction in functional connectivity (Phase Lag Index) in PDD compared to PD.

## 1 Introduction

Parkinson's disease (PD) is a progressive neurodegenerative disorder associated with both motor and a variety of non-motor symptoms including cognitive dysfunction and dementia. Parkinson's disease related dementia (PDD) develops in up to 80% of PD patients (Aarsland and Kurz, 2010; Reid et al., 2011) and is characterized by executive dysfunction and memory deficits, often in combination with visual hallucinations (Emre et al., 2007). The pathophysiological mechanisms of cognitive dysfunction and dementia in PDD are still poorly understood. A loss of nigrostriatal and corticopetal dopaminergic, serotonergic and noradrenergic projection systems may be involved (Cash et al., 1987; Kuhl et al., 1996; Mattila et al., 2001; Rinne et al., 1989). Additional mechanisms that may contribute to the development of dementia in PD are degeneration of cholinergic cortical projections and/or local cortical Lewy body- and tau-pathology (Bosboom et al., 2004). The involvement of multiple projection systems is supported by the neuropathological findings by Braak et al. (2003), indicating that PD specific brain pathology extends beyond the nigrostriatal dopaminergic system. Electroencephalography (EEG) and magnetoencephalography (MEG) can be used to directly study neuronal-network activity. There is now increasing evidence that oscillatory brain activity is related to normal and disturbed cognitive function (Uhlhaas and Singer, 2006). Several MEG and EEG studies have demonstrated a slowing of background oscillatory activity in PDD patients (Bosboom et al., 2006; Kotini et al., 2005; Neufeld et al., 1988, 1994; Soikkeli et al., 1991; Stoffers et al., 2007; Tanaka et al., 2000), and a recent study by Klassen et al. (2011) showed that EEG parameters have a predictive value for the development of dementia in non-demented PD patients.

In addition to changes in oscillatory brain activity, altered functional coupling between distributed brain areas has also been observed in PD. Stoffers et al. (2008) showed that the interregional synchronization between different brain areas, referred to as functional connectivity, is increased in theta (4–8 Hz), alpha (8–13 Hz) and beta (13–30 Hz) bands in non-demented PD patients compared to healthy individuals. In contrast, reduced functional coupling in delta (0.5–4 Hz), theta and alpha rhythms was found in PD related dementia, which was most pronounced for inter-temporal and fronto-temporal functional connections (Bosboom et al., 2009).

Considering the involvement of multiple neurotransmitter systems in PD (Braak et al., 2003), it has been speculated that the widespread increase in functional coupling in the early stages of the disease may result from degeneration of ascending brainstem monoaminergic projections, while on the other hand degeneration of the cholinergic system may contribute to the fronto-temporal (alpha band) and centro-parietal (gamma band) reductions in functional coupling in more advanced PD related dementia (Bosboom et al., 2006; Braak et al., 2003, 2005). Together, these results suggest that there are stage-specific, partly opposite, patterns of change in functional coupling in PD. Nevertheless, the exact pathophysiological mechanisms in the development of PD related dementia still need to be elucidated.

A limitation of our previous MEG studies (Bosboom et al., 2009; Stoffers et al., 2008) is that the analyses were performed at the sensor level, i.e. functional connectivity was estimated on the basis of the extracranial recordings directly. Relating the functional sensor based data to its exact underlying anatomical substrate is problematic, hampering the interpretation of these results. To overcome this problem, we recently developed a new analysis technique (Hillebrand et al., 2011), projecting sensor-based data onto an atlas-based source space using beamforming, providing a detailed anatomical mapping of cortical rhythms for 68 regions of interest corresponding to Brodmann areas. A further limitation of sensor-level analysis is that most measures of functional connectivity are influenced by the effects of volume conduction and field spread, such that multiple recording sites pick-up signals from a single source and multiple sources can project to the same recording site, which may result in erroneous estimates of functional connectivity (García Domínguez et al., 2012; Schoffelen and Gross, 2009). The above-mentioned approach of projecting the data into source space in itself does not solve this problem (Hillebrand et al., 2011). However, the effects of volume conduction can be removed in either signal space or source space by estimating functional connectivity using the phase lag index (PLI), which is a measure that quantifies the consistency of non-zero phase differences between two signals (Stam et al., 2007).

The aim of the present study was to expand on our previous studies using an atlas-based beamformer in combination with PLI to shed more light on the anatomical distribution of changes in cortical rhythms and functional interactions. To this end, spectral power and functional connectivity profiles were estimated on the basis of resting-state MEG recordings, and the profiles for groups of demented and non-demented PD patients were subsequently compared.

## 2 Materials and methods

### 2.1 Study population/subjects

The present study involved two groups of subjects, previously described and analysed in studies on PD for which approval was obtained from the medical ethics committee of the VU University Medical Center (Bosboom et al., 2006). The groups consisted of PD patients without dementia (PD; N = 13; 7♀/6♂) and PD patients with dementia (PDD; N = 13; 5♀/8♂). The demographic characteristics of the two study groups are listed in Table 1. Disease duration was not significantly different between demented (mean 11.2 years) and non-demented patients (mean 9.7 years). Demented patients had significantly higher UPDRS III motor scores (mean 23.9) compared to non-demented PD patients (mean 16.2). All participants gave written informed consent prior to participation.

### 2.2 MEG procedures

MEG data were acquired using a 151-channel whole head MEG system (CTF Systems Inc., Port Coquitlam, BC, Canada). Patients were seated in a magnetically shielded room (Vacuum-schmelze GmbH, Hanau, Germany). All MEG recordings were acquired in the morning. Patients were asked to skip their first morning dose of dopaminergic medication (practically defined “OFF”) for MEG registration. MEG was recorded in an eyes closed resting-state condition (EC) followed by an eyes open resting-state condition (EO). In the latter condition, patients were asked to avoid ocular movement and eye blinks. More detailed information regarding the MEG data acquisition can be found in Bosboom et al. (2006). In addition to MEG, an anatomical MRI of the head was obtained for each subject (1 T, Impact, Siemens, Erlangen, Germany), with an in-plane resolution of 1 mm and slice thickness of 1.5 mm. Vitamin E capsules were placed at the same anatomical landmarks where head-localisation coils were placed during the MEG scan; the pre-auricular points and the nasion. The MEG and MRI data were co-registered by aligning these two corresponding sets of fiducial markers.

### 2.3 Analysis

The analysis procedure is described in detail in Hillebrand et al. (2011). To summarise, the main idea is that beamforming is used to project the MEG sensor signals to a set of time-series of neuronal activation for a total of 68 atlas-based regions of interest (ROIs; Appendix A and Supplementary material). Twenty artefact-free data-segments of 1024 samples were selected from the ROI time-series after careful visual inspection (MMP). Time-series of neuronal activation were computed for the 5 classical EEG bands; delta (0.5–4 Hz), theta (4–8 Hz), alpha (8–13 Hz), beta (13–30 Hz) and gamma (30–48 Hz), resulting in a total of 5 sets (one for each frequency band) of 68 time-series for further analysis. For each patient group and each frequency band the (i) mean (over trials and patients) relative oscillatory power was computed for each ROI; (ii) functional connectivity between ROIs was estimated using the phase lag index (PLI). PLI discards the effects of volume conduction and field spread through quantification of the (non-zero lag) phase coupling between two oscillatory signals (Stam et al., 2007). The mean (over trials and patients) PLI was computed for all possible pairs of ROIs (i.e. the (mean) full 68 × 68 adjacency matrix was estimated). Subsequently, two analyses were performed: i) for each ROI the mean PLI between that ROI and all other ROIs was computed, which is a measure of the overall connectivity of a region (also known as the weighted degree or node strength in terms of graph theory (Rubinov and Sporns, 2010)); ii) the details of the connectivity patterns between pairs of ROIs were examined.

The mean relative power and PLI values for each ROI were compared between the PD and PPD groups by means of permutation analysis (Nichols and Holmes, 2002), where a null distribution for between-group differences is derived by permuting group assignment and calculating a t-statistic after each permutation. To correct for multiple comparisons, the maximum t-value across ROIs for each permutation was used to construct a distribution of maximum t-values for 1000 permutations. The threshold at the 0.05 significance level for this distribution of maximum values was determined and subsequently applied to determine whether observed t-values at the individual ROIs reached significance.

Similarly, permutation analysis was used to determine whether there were significant differences in the PLI values between pairs of ROIs when comparing the PD and PDD groups. The threshold was set at a 0.05 significance level to determine whether PLI values between pairs of ROIs were weaker or stronger in PDD.

## 3 Results

### 3.1 Power spectral analysis

**Supplementary Fig. S1:**
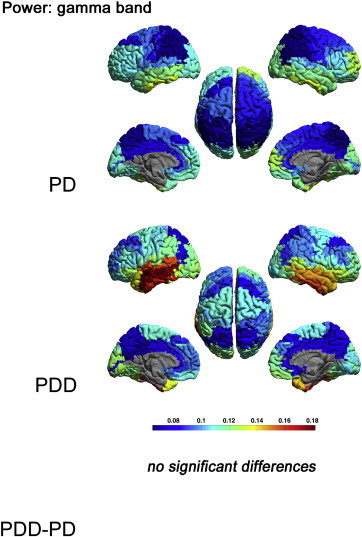
Relative power, shown as a colour-coded map on a template mesh, for the PD and PDD groups in the gamma band. No significant differences were found in the gamma band between groups using permutation analysis (global-threshold). The left hemisphere is shown on the left.

**Fig. 1:**
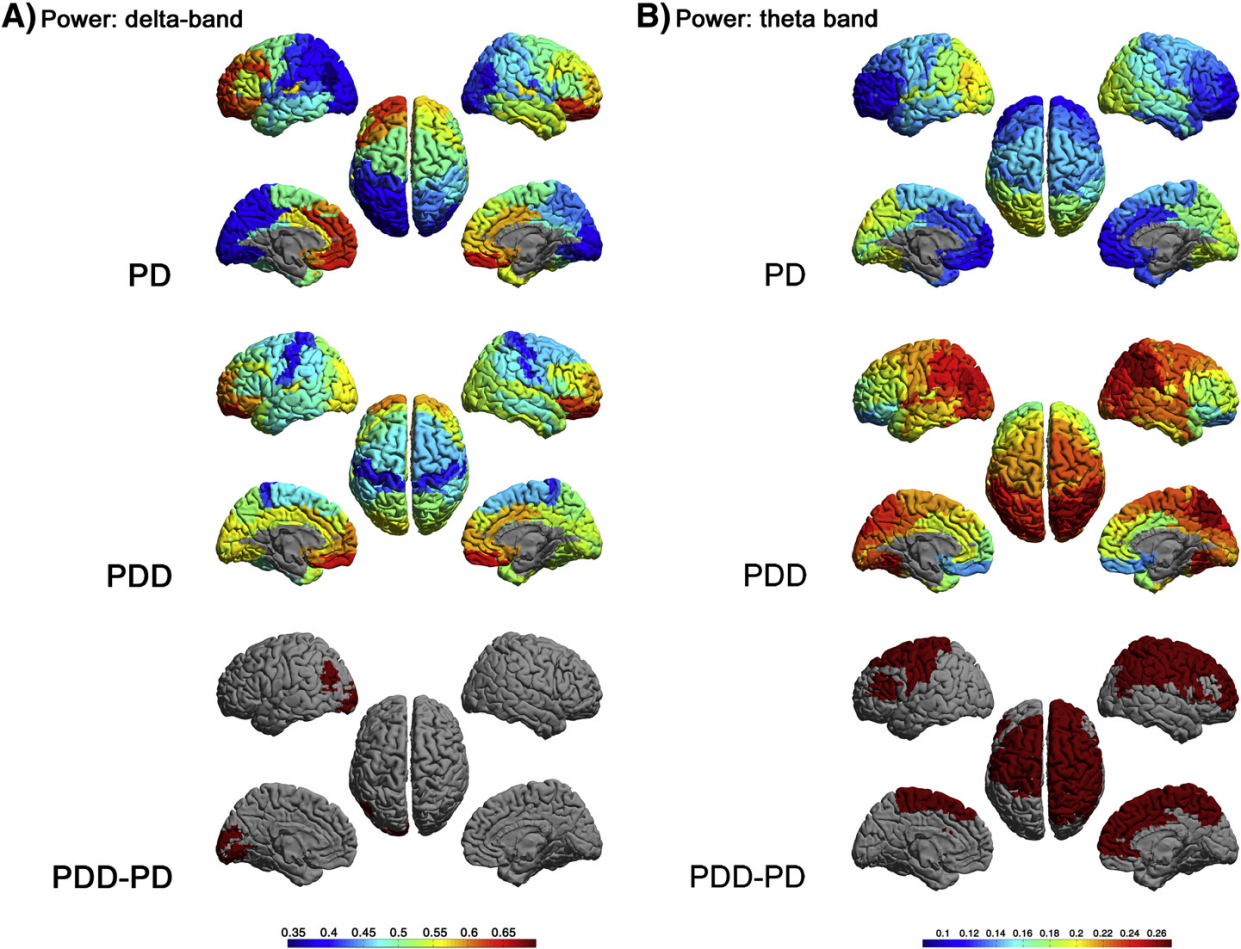

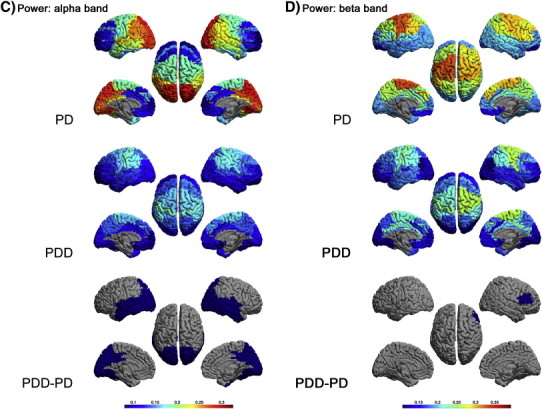
Relative power, shown as a colour-coded map on a template mesh, for the PD (top panel) and PDD groups (middle panel) in respectively the delta (A), theta (B), alpha (C) and beta (D) bands. The colour bar denotes the relative power in the top and middle panels. The lower panel shows the significant differences (positive in red; negative in blue) in corresponding bands between the two groups as determined using permutation analysis (global-threshold). The left hemisphere is shown on the left.

Figs. 1 and S1 display respectively the relative delta, theta, alpha, beta and gamma power for all ROIs in each group, as well as the differences between groups. The nomenclature for the ROIs is given in Appendix A and the Supplementary material.

Figs. 1 and S1 display respectively the relative delta, theta, alpha, beta and gamma power for all ROIs in each group, as well as the differences between groups. The nomenclature for the ROIs is given in Appendix A and the Supplementary material.

Compared to PD, the PDD patients had more delta power in the left angular gyrus (BA 39L) and the left secondary visual cortex (BA 18L) (Fig. 1A).

In addition, the PDD patients had (Fig. 1B) significantly more theta power in parietal areas: primary and somatosensory association cortex, and right angular and supramarginal gyrus (BA 1, 2, 3, 5, 7R, 39R, 40R) and frontal areas: primary motor cortex, premotor cortex, supplementary motor area, frontal cortex including frontal eye fields and right dorsolateral prefrontal cortex, right anterior prefrontal cortex, right ventral (dorsal) anterior cingulate, anterior cingulate cortex, pars triangularis and left dorsolateral prefrontal cortex (BA 4, 6, 8, 9R, 10R, 24R, 32R, 33, 45, 46L).

Compared to PD patients (Fig. 1C), PDD patients had less alpha power in occipital areas: primary, secondary and associative visual cortex (BA 17, 18, 19), temporal areas: right inferior temporal gyrus, middle temporal gyrus, right superior temporal gyrus, fusiform gyrus, primary and auditory association cortex and right primary gustatory cortex (BA 20,21, 22, 37,41,42, 43R), parietal areas: ventral posterior cingulate, dorsal posterior cingulate cortex, and somatosensory association cortex (BA 23, 31, 39, 7).

Beta power was significantly lower in the right frontal areas: pars opercularis, triangularis and dorsolateral prefrontal cortex (BA 44R, 45R, 46R) for PDD patients compared to PD patients (Fig. 1D).

The power in the gamma band did not differ significantly between groups (Supplementary material, Fig. S1).

The power in the gamma band did not differ significantly between groups (Supplementary material, Fig. S1).

### 3.2 Mean connectivity per and between regions

**Supplementary Fig. S2:**
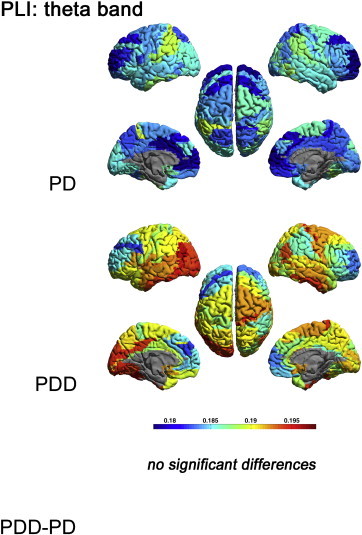
Mean PLI for each ROI, shown as a colour-coded map on a template mesh, for the PD and PDD groups in the theta band. No significant differences were found in the theta band between groups using permutation analysis (global-threshold). The left hemisphere is shown on the left.

**Supplementary Fig. S3:**
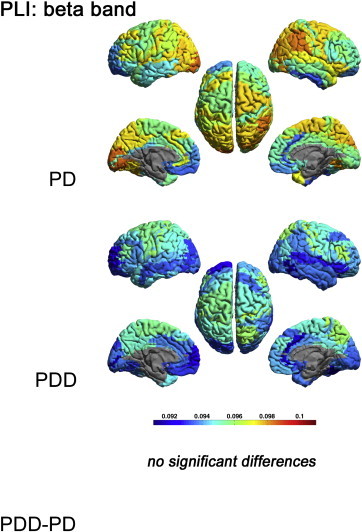
Mean PLI for each ROI, shown as a colour-coded map on a template mesh, for the PD and PDD groups in the beta band. No significant differences were found in the beta band between groups using permutation analysis (global-threshold). The left hemisphere is shown on the left.

**Supplementary Fig. S4:**
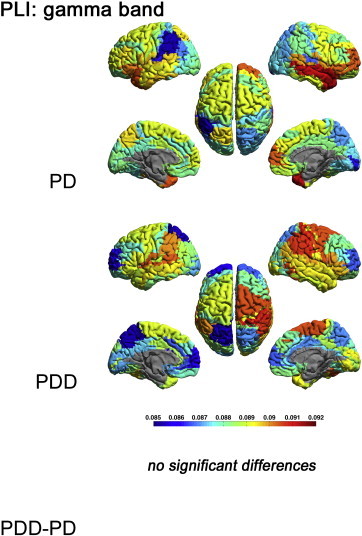
Mean PLI for each ROI, shown as a colour-coded map on a template mesh, for the PD and PDD groups in the gamma band. No significant differences were found in the gamma band between groups using permutation analysis (global-threshold). The left hemisphere is shown on the left.

**Fig. 2:**
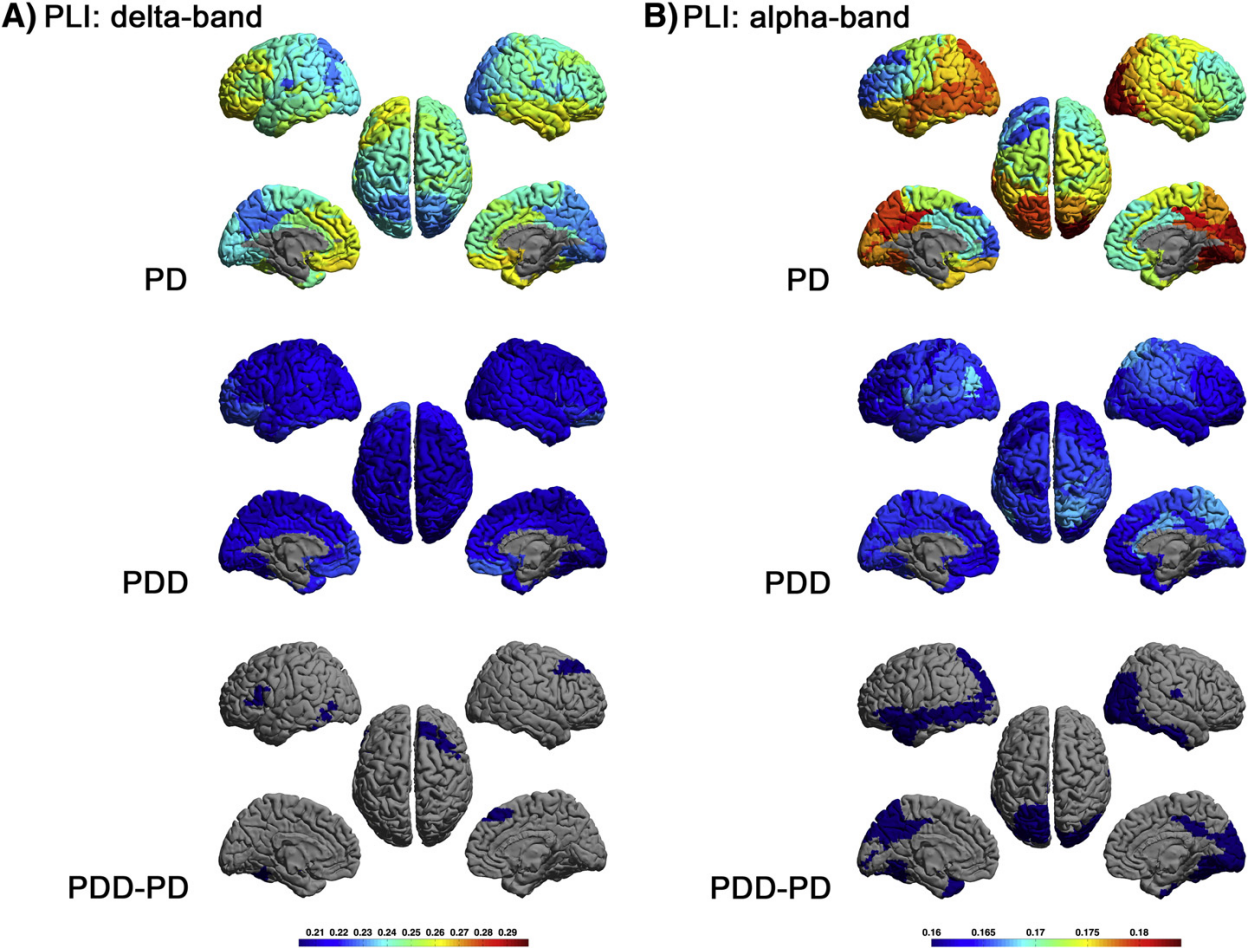
Mean PLI for each ROI, shown as a colour-coded map on a template mesh, for the PD (top panel) and PDD (middle panel) groups in respectively the delta (A) and alpha (B) bands. The colour bar denotes the PLI in the top and middle panels. The lower panel shows the significant differences (positive in red; negative in blue) in the corresponding bands between the two groups as determined using permutation analysis (global-threshold). The left hemisphere is shown on the left.

Figs. 2 and S2–S4 display the mean PLI between each ROI and all other ROIs in respectively the delta, theta, alpha, beta and gamma bands for each group, as well as the differences between groups.

Figs. 2 and S2–S4 display the mean PLI between each ROI and all other ROIs in respectively the delta, theta, alpha, beta and gamma bands for each group, as well as the differences between groups.

Compared to PD patients (Fig. 2A), the delta band PLI in PDD patients was significantly lower in the left fusiform gyrus (BA 37L) and frontal areas: right frontal cortex, including the frontal eye fields and left pars triangularis (BA 8R, 45L).

The alpha band PLI was significantly lower in the occipital areas: primary and associative visual cortex (BA 17, 18R, 19), temporal areas: right inferior temporal gyrus, left middle temporal gyrus, fusiform gyrus and left temporopolar area (BA 20R, 21L, 37, 38L) and parietal areas: left somatosensory association cortex, dorsal posterior cingulate cortex, right angular gyrus, right primary gustatory cortex and left inferior prefrontal cortex (BA 7L, 31, 39R, 43R, 47L) (Fig. 2B).

The theta, beta and gamma band PLI did not differ significantly between groups (Supplementary material, Figs. S2–S4).

The theta, beta and gamma band PLI did not differ significantly between groups (Supplementary material, Figs. S2–S4).

Supplementary Table S1 summarises all results between groups across regions and frequency bands.

Supplementary Table S1 summarises all results between groups across regions and frequency bands.

**Fig. 3:**
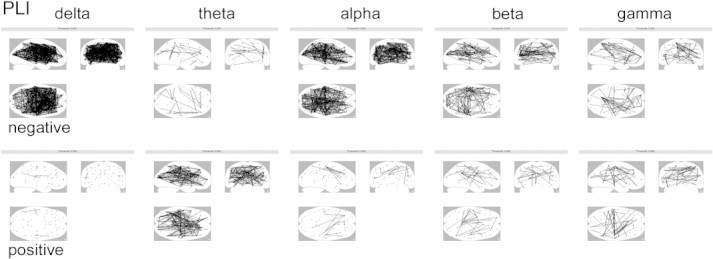
Differences (negative = weaker, positive = stronger) in region-to-region connectivity in the PDD group compared to the PD group in respectively the delta, theta, alpha, beta and gamma bands, displayed on a glass-brain threshold: p < 0.05, uncorrected.

Fig. 3 displays the results of a detailed analysis of all differences in connectivity between pairs of ROIs between the PD and PDD groups. Theta band connectivity between pairs of ROIs was diffusely stronger in PDD patients compared to PD patients. In addition, connectivity was generally weaker in PDD patients compared to PD patients in the delta, alpha and, to a lesser extent, beta bands. In the gamma band, connectivity was stronger for some and weaker for other pairs of ROIs in the PDD group.

## 4 Discussion

This is the first study to use MEG recordings projected to an atlas-based source space to provide a 3D anatomical view of differences in oscillatory power and functional connectivity between PD and PDD patients. In addition, the estimation of functional connectivity was based upon the PLI, a measure that rigorously avoids any biases due to volume conduction or field spread. Our main findings are: i) stronger activation in PDD compared to PD in the delta and theta bands, in respectively the parieto-occipital and fronto-parietal areas; ii) in the alpha and beta bands, less power was found in PDD patients compared to PD in respectively the occipito-parieto-temporal and frontal areas; iii) functional connectivity was weaker in the delta and alpha bands in respectively the fronto-temporal and occipito-parieto-temporal areas; iv) a more detailed analysis revealed that functional connectivity between pairs of ROIs was generally weaker in PDD patients compared to PD patients in the delta, alpha and to a lesser extent beta bands, whereas functional connectivity between pairs of ROIs was stronger in the theta band. In the gamma band, connectivity was stronger for some and weaker for other pairs of ROIs in the PDD group.

Our observations are in line with previous studies (Bosboom et al., 2006; Neufeld et al., 1988, 1994; Olde Dubbelink et al., 2012; Soikkeli et al., 1991; Tanaka et al., 2000) revealing a slowing of rhythmic brain activity in PDD patients compared to PD patients. However, in these previous studies, only an approximate anatomical distribution of changes in neuronal activity could be given. The present method enabled us to define the exact anatomical areas where neuronal activity was different. This advantage is also visible in the functional connectivity measures, revealing the exact Brodmann areas for which functional coupling changes in PDD. Our present results differ somewhat from previous findings by our group (Hillebrand et al., 2011) showing weaker functional connectivity over fronto-temporal areas in the alpha band and over centro-parietal areas in the gamma band in PDD patients compared to PD patients. We found no significant differences in the gamma band for the average connectivity, whereas the alpha band differences in our present analysis were not only restricted to temporal areas, but also involved more posterior brain regions. These discrepancies can at least partly be explained by our previous methodological approach (Bosboom et al., 2009), which focused on functional connections for 10 (arbitrarily chosen) clusters of extracranial sensors. This sensor-based approach was based upon the assumption that each cluster would roughly correspond to a particular anatomical region. Given the effects of both source orientation and field spread on the distribution of the extracranial magnetic field, linking their functional results to its underlying anatomical substrate, however, is challenging. Another possible explanation for the differences between both studies is that synchronization likelihood (SL) was used as a measure of functional connectivity in our previous study. Most measures of functional connectivity, including SL, are influenced by the effects of volume conduction resulting in erroneous estimates of functional connectivity (García Domínguez et al., 2012; Schoffelen and Gross, 2009; Stam et al., 2007). We now used the PLI as a measure of functional connectivity to avoid such biases.

In the PDD patients, many neocortical areas showed prominent activity in the lower frequency bands (Fig. 1A,B). The increase in slow wave activity in PDD patients compared to non-demented PD patients may be a reflection of the different trajectories of development of the degenerative process in the two groups. This is in line with recent neuropathological data (Halliday and McCann, 2010; Halliday et al., 2008; Selikhova et al., 2009) suggesting distinct phenotypes with different trajectories of development.

Cholinergic drugs and dopamine replacement therapy can influence brain activity. In the present study, however, patients were not using cholinesterase inhibitors. Furthermore, to minimise the modulatory effects of dopamine replacement therapy we performed the MEG registrations in a practically defined OFF state. Nevertheless, we cannot fully exclude the possibility that differences in the medication regime between the PD and PDD groups may have contributed to the observed differences in oscillatory functional networks.

It is tempting to speculate that the observed loss of functional connectivity in particularly the fronto-temporal regions (Fig. 2) in PD related dementia might be related to the cognitive dysfunction as frontal and temporal regions are known to be important in respectively executive and memory functions. This hypothesis is supported by observations in AD (Stam et al., 2006) and dementia with Lewy bodies (DLB) (Franciotti et al., 2006) showing similar changes in functional connectivity, suggesting that reductions in functional connectivity in PDD may be related to degeneration of the cholinergic system. Another possible cause of the observed changes in power and functional connectivity might be loss of structural connections due to cortical atrophy and/or loss of cortico-cortical axons. Similar to AD, cortical atrophy is prominent in PD related dementia (Beyer et al., 2007; Burton et al., 2002; Camicioli et al., 2003; Ibarretxe-Bilbao et al., 2008; Junque et al., 2005; Tam et al., 2005). An fMRI study in a group of AD patients demonstrated an association between fMRI coherence and cortical atrophy (He et al., 2007). As a consequence, exploring the contribution of local cortical changes to the alterations of functional connectivity in PDD is an interesting topic for future research.

AD as well as PDD is thought to be a disconnection syndrome which is explained as a failure of network function due to interaction failure among the regions of the network (Bokde et al., 2009; Braak et al., 2004; Cronin-Golomb, 2010; Cronin-Golomb and Braun, 1997). In AD, this hypothesis is supported by multiple lines of research including neurophysiological studies (Stam et al., 2003). In AD, the breakdown is thought to be the result of chronically progressive neuropathology with underlying molecular mechanisms leading to neuronal and synaptic dysfunction and ultimately to neuronal loss. Similar mechanisms are thought to be the underlying cause of the disconnection hypothesis in PD. In PD, this hypothesis is supported by neuropsychological and MRI studies (Cronin-Golomb, 2010; Kostic and Filippi, 2011). Our present study shows an extensive reduction in binding between different regions supporting the disconnection hypothesis in PDD with neurophysiologic measures.

In conclusion, the present study is the first to demonstrate, in an anatomical framework, the slowing of brain rhythms in PD related dementia compared to non-demented PD. In addition, this study shows a widespread reduction in functional connectivity between different regions in PDD compared to non-demented PD. To further elucidate the link between reduced functional connectivity and cognitive performance in PDD patients, an atlas-based beamformer approach could also be used in future network analyses.

The following are the supplementary data related to this article.

Supplementary Table S1 Overview of all significant results (red/+ = increase and blue/− = decrease) in relative power and mean PLI in different regions and frequency bands in the PDD group compared to the PD group.

Supplementary material.

Supplementary data to this article can be found online at http://dx.doi.org/10.1016/j.nicl.2012.11.007.

## Figures and Tables

**Table A1:** List of Brodmann areas, and the labels, that were used. L denotes the left hemisphere, and R denotes the right hemisphere. See the movie in the Supplementary material for the location of each region on a template mesh.

Index	ROI label	Index	ROI label
1	BA 1: primary somatosensory cortex (L)	35	BA 37: fusiform gyrus (L)
2	BA 1: primary somatosensory cortex (R)	36	BA 37: fusiform gyrus (R)
3	BA 10: anterior prefrontal cortex (L)	37	BA 38: temporopolar area (L)
4	BA 10: anterior prefrontal cortex (R)	38	BA 38: temporopolar area (R)
5	BA 11: orbitofrontal cortex (L)	39	BA 39: angular gyrus (L)
6	BA 11: orbitofrontal cortex (R)	40	BA 39: angular gyrus (R)
7	BA 17: primary visual cortex (L)	41	BA 4: primary motor cortex (L)
8	BA 17: primary visual cortex (R)	42	BA 4: primary motor cortex (R)
9	BA 18: secondary visual cortex (L)	43	BA 40: supramarginal gyrus (L)
10	BA 18: secondary visual cortex (R)	44	BA 40: supramarginal gyrus (R)
11	BA 19: associative visual cortex (L)	45	BA 41: primary and auditory association cortex (L)
12	BA 19: associative visual cortex (R)	46	BA 41: primary and auditory association cortex (R)
13	BA 2: primary somatosensory cortex (L)	47	BA 42: primary and auditory association cortex (L)
14	BA 2: primary somatosensory cortex (R)	48	BA 42: primary and auditory association cortex (R)
15	BA 20: inferior temporal gyrus (L)	49	BA 43: primary gustatory cortex (L)
16	BA 20: inferior temporal gyrus (R)	50	BA 43: primary gustatory cortex (R)
17	BA 21: middle temporal gyrus (L)	51	BA 44: pars opercularis (L)
18	BA 21: middle temporal gyrus (R)	52	BA 44: pars opercularis (R)
19	BA 22: superior temporal gyrus (L)	53	BA 45: pars triangularis (L)
20	BA 22: superior temporal gyrus (R)	54	BA 45: pars triangularis (R)
21	BA 23: ventral posterior cingulate (L)	55	BA 46: dorsolateral prefrontal cortex (L)
22	BA 23: ventral posterior cingulate (R)	56	BA 46: dorsolateral prefrontal cortex (R)
23	BA 24: ventral anterior cingulate (L)	57	BA 47: inferior prefrontal gyrus (L)
24	BA 24: ventral anterior cingulate (R)	58	BA 47: inferior prefrontal gyrus (R)
25	BA 25: ventromedial prefrontal cortex (L)	59	BA 5: somatosensory association cortex (L)
26	BA 25: ventromedial prefrontal cortex (R)	60	BA 5: somatosensory association cortex (R)
27	BA 3: primary somatosensory cortex (L)	61	BA 6: premotor cortex and supplementary motor area (L)
28	BA 3: primary somatosensory cortex (R)	62	BA 6: premotor cortex and supplementary motor area (R)
29	BA 31: dorsal posterior cingulate cortex (L)	63	BA 7: somatosensory association cortex (L)
30	BA 31: dorsal posterior cingulate cortex (R)	64	BA 7: somatosensory association cortex (R)
31	BA 32: dorsal anterior cingulate cortex (L)	65	BA 8: frontal cortex including frontal eye fields (L)
32	BA 32: dorsal anterior cingulate cortex (R)	66	BA 8: frontal cortex including frontal eye fields (R)
33	BA 33: anterior cingulate cortex (L)	67	BA 9: dorsolateral prefrontal cortex (L)
34	BA 33: anterior cingulate cortex (R)	68	BA 9: dorsolateral prefrontal cortex (R)

**Table 1:** Mean and standard deviation (SD) of the demographic characteristics for the PD and PD groups.

	PDD	SD	PD	SD
Sex (M/F)	8/5		6/7	
Age (years)	74.4	4.9	71.7	5.1
Disease duration (years)	11.2	4.0	9.69	4.5
CAMCOG (0–107)	71.5	12	96.0	5
UPDRS III off (0–108)	23.9	6	16.2	3
H&Y stage off (0–5)	2.9		2.5	
