# Supplementary material for: A three dimensional anatomical view of oscillatory resting-state activity and functional connectivity in Parkinson's disease related dementia: An MEG study using atlas-based beamforming
Source: Neuroimage Clin. 2012 Nov 17;2:95–102. doi: 10.1016/j.nicl.2012.11.007 (PMC3777782; doi:10.1016/j.nicl.2012.11.007)
Supplement: Supplementary Table S1 — Overview of all significant results (red/+ = increase and blue/− = decrease) in relative power and mean PLI in different regions and frequency bands in the PDD group compared to the PD group. [file mmc1.doc]

**Table 2**

| **ROI label** | **Power** | | | | | **PLI** | | | | |
| --- | --- | --- | --- | --- | --- | --- | --- | --- | --- | --- |
|  | **delta** | **theta** | **alpha** | **beta** | **gamma** | **delta** | **theta** | **alpha** | **beta** | **gamma** |
| BA 1: Primary Somatosensory Cortex (L) |  | + |  |  |  |  |  |  |  |  |
| BA 1: Primary Somatosensory Cortex (R) |  | + |  |  |  |  |  |  |  |  |
| BA 10: Anterior Prefrontal Cortex (L) |  |  |  |  |  |  |  |  |  |  |
| BA 10: Anterior Prefrontal Cortex (R) |  | + |  |  |  |  |  |  |  |  |
| BA 11: Orbitofrontal Cortex (L) |  |  |  |  |  |  |  |  |  |  |
| BA 11: Orbitofrontal Cortex (R) |  |  |  |  |  |  |  |  |  |  |
| BA 17: Primary Visual Cortex (L) |  |  | - |  |  |  |  | - |  |  |
| BA 17: Primary Visual Cortex (R) |  |  | - |  |  |  |  | - |  |  |
| BA 18: Secondary Visual Cortex (L) | + |  | - |  |  |  |  |  |  |  |
| BA 18: Secondary Visual Cortex (R) |  |  | - |  |  |  |  |  |  |  |
| BA 19: Associative Visual Cortex (L) |  |  | - |  |  |  |  | - |  |  |
| BA 19: Associative Visual Cortex (R) |  |  | - |  |  |  |  | - |  |  |
| BA 2: Primary Somatosensory Cortex (L) |  | + |  |  |  |  |  |  |  |  |
| BA 2: Primary Somatosensory Cortex (R) |  | + |  |  |  |  |  |  |  |  |
| BA 20: Inferior Temporal Gyrus (L) |  |  | - |  |  |  |  |  |  |  |
| BA 20: Inferior Temporal Gyrus (R) |  |  | - |  |  |  |  | - |  |  |
| BA 21: Middle Temporal Gyrus (L) |  |  | - |  |  |  |  | - |  |  |
| BA 21: Middle Temporal Gyrus (R) |  |  | - |  |  |  |  |  |  |  |
| BA 22: Superior Temporal Gyrus (L) |  |  | - |  |  |  |  |  |  |  |
| BA 22: Superior Temporal Gyrus (R) |  |  | - |  |  |  |  |  |  |  |
| BA 23: Ventral Posterior Cingulate (L) |  |  | - |  |  |  |  |  |  |  |
| BA 23: Ventral Posterior Cingulate (R) |  |  | - |  |  |  |  |  |  |  |
| BA 24: Ventral Anterior Cingulate (L) |  |  |  |  |  |  |  |  |  |  |
| BA 24: Ventral Anterior Cingulate (R) |  | + |  |  |  |  |  |  |  |  |
| BA 25: Ventromedial Prefrontal Cortex (L) |  |  |  |  |  |  |  |  |  |  |
| BA 25: Ventromedial Prefrontal Cortex (R) |  |  |  |  |  |  |  |  |  |  |
| BA 3: Primary Somatosensory Cortex (L) |  | + |  |  |  |  |  |  |  |  |
| BA 3: Primary Somatosensory Cortex (R) |  | + |  |  |  |  |  |  |  |  |
| BA 31: Dorsal Posterior Cingulate Cortex (L) |  |  | - |  |  |  |  | - |  |  |
| BA 31: Dorsal Posterior Cingulate Cortex (R) |  |  | - |  |  |  |  | - |  |  |
| BA 32: Dorsal Anterior Cingulate Cortex (L) |  |  |  |  |  |  |  |  |  |  |
| BA 32: Dorsal Anterior Cingulate Cortex (R) |  | + |  |  |  |  |  |  |  |  |
| BA 33: Anterior Cingulate Cortex (L) |  | + |  |  |  |  |  |  |  |  |
| BA 33: Anterior Cingulate Cortex (R) |  | + |  |  |  |  |  |  |  |  |
| BA 37: Fusiform Gyrus (L) |  |  | - |  |  | - |  | - |  |  |
| BA 37: Fusiform Gyrus (R) |  |  | - |  |  |  |  | - |  |  |
| BA 38: Temporopolar Area (L) |  |  |  |  |  |  |  | - |  |  |
| BA 38: Temporopolar Area (R) |  |  |  |  |  |  |  |  |  |  |
| BA 39: Angular Gyrus (L) | + |  | - |  |  |  |  |  |  |  |
| BA 39: Angular Gyrus (R) |  | + | - |  |  |  |  | - |  |  |
| BA 4: Primary Motor Cortex (L) |  | + |  |  |  |  |  |  |  |  |
| BA 4: Primary Motor Cortex (R) |  | + |  |  |  |  |  |  |  |  |
| BA 40: Supramarginal Gyrus (L) |  |  |  |  |  |  |  |  |  |  |
| BA 40: Supramarginal Gyrus (R) |  | + |  |  |  |  |  |  |  |  |
| BA 41: Primary and Auditory Association Cortex (L) |  |  | - |  |  |  |  |  |  |  |
| BA 41: Primary and Auditory Association Cortex (R) |  |  | - |  |  |  |  |  |  |  |
| BA 42: Primary and Auditory Association Cortex (L) |  |  | - |  |  |  |  |  |  |  |
| BA 42: Primary and Auditory Association Cortex (R) |  |  | - |  |  |  |  |  |  |  |
| BA 43: Primary Gustatory Cortex (L) |  |  |  |  |  |  |  |  |  |  |
| BA 43: Primary Gustatory Cortex (R) |  |  | - |  |  |  |  | - |  |  |
| BA 44: Pars Opercularis (L) |  |  |  |  |  |  |  |  |  |  |
| BA 44: Pars Opercularis (R) |  |  |  | - |  |  |  |  |  |  |
| BA 45: Pars Triangularis (L) |  | + |  |  |  | - |  |  |  |  |
| BA 45: Pars Triangularis (R) |  | + |  | - |  |  |  |  |  |  |
| BA 46: Dorsolateral Prefrontal Cortex (L) |  | + |  |  |  |  |  |  |  |  |
| BA 46: Dorsolateral Prefrontal Cortex (R) |  |  |  | - |  |  |  |  |  |  |
| BA 47: Inferior Prefrontal Gyrus (L) |  |  |  |  |  |  |  | - |  |  |
| BA 47: Inferior Prefrontal Gyrus(R) |  |  |  |  |  |  |  |  |  |  |
| BA 5: Somatosensory Association Cortex (L) |  | + |  |  |  |  |  |  |  |  |
| BA 5: Somatosensory Association Cortex (R) |  | + |  |  |  |  |  |  |  |  |
| BA 6: Premotor Cortex and Supplementary Motor Area (L) |  | + |  |  |  |  |  |  |  |  |
| BA 6: Premotor Cortex and Supplementary Motor Area (R) |  | + |  |  |  |  |  |  |  |  |
| BA 7: Somatosensory Association Cortex (L) |  |  | - |  |  |  |  | - |  |  |
| BA 7: Somatosensory Association Cortex (R) |  | + | - |  |  |  |  |  |  |  |
| BA 8: Frontal Cortex including Frontal Eye (L)(lFields (L) |  | + |  |  |  |  |  |  |  |  |
| BA 8: Frontal Cortex including Frontal Eye Fields (R) |  | + |  |  |  | - |  |  |  |  |
| BA 9: Dorsolateral Prefrontal Cortex (L) |  |  |  |  |  |  |  |  |  |  |
| BA 9: Dorsolateral Prefrontal Cortex (R) |  | + |  |  |  |  |  |  |  |  |
